# Prevalence difference of *Helicobacter pylori* infection between Tibetan and Han ethnics: The protocol of a meta-analysis on epidemiologic studies

**DOI:** 10.1097/MD.0000000000018566

**Published:** 2019-12-27

**Authors:** Dan Bai, An-Mo Wang, Kai Liu, Si-Yu Duan, Wei-Han Zhang, Jian-Kun Hu, Xin-Zu Chen

**Affiliations:** a Department of Gastrointestinal Surgery & Laboratory of Gastric Cancer, West China Hospital, Sichuan University, Chengdu; b Faculty of Clinical Medicine, West China Medical School, Sichuan University, Chengdu; c Department of Gastrointestinal and Hernia Surgery, West China Yibin Hospital, Sichuan University, Yibin; d Department of General Surgery, West China Longquan Hospital, Sichuan University, Chengdu, China.

**Keywords:** epidemiology, *Helicobacter pylori*, meta-analysis, prevalence, Tibetan

## Abstract

**Objectives:**

*Helicobacter pylori* (Hp) is an identified carcinogenic pathogen of human gastric cancer. China is not only one of the countries with high incidence and mortality of gastric cancer, but also a high infection area of Hp. As a multi-ethnic country, China may have a diverse prevalence of Hp infection among ethnics. This meta-analysis tends to compare the prevalence of Hp infection between Tibetan and Han ethnics, the results may provide evidence for targeted screening and eradication of Hp in China.

**Methods:**

The following databases will be searched: PubMed, Web of Science, Technology Periodical Database (VIP), China National Knowledge infrastructure (CNKI), and WanFang databases. Studies which reported the prevalence of Hp infection between Tibetans and Hans in China are eligible. Two reviewers will independently screen studies, extract data and assess the risk of bias of included studies. The prevalence of Hp infection between Tibetan and Han ethnics will be compared by meta-analysis. Heterogeneity tests and meta-analyses will be conducted using RevMan 5.3 and Stata 12.0 softwares. Meanwhile, subgroup analysis, publication bias and sensitivity analysis evaluation will be performed where applicable.

**Results:**

This study will be reported in compliance with the PRISMA statement.

This systematic review will not be submitted for any ethical approval since no privacy health information will be included. The findings will be published through peer-reviewed publications or conference presentations.

**Prospero Registration Number:**

CRD42019121192.

**Conclusions:**

Our study will provide us evidence for tailored strategy and robustness of Hp screening and eradication among Tibetans.

**Keypoints**

- As far as we know, there is no comprehensive review on the prevalence of Hp infection between Tibetan and Han ethnics.
- A tailored strong screening and eradication strategy for Hp infection need consider among Tibetans.
- Although multiple databases will be comprehensively searched, the limited Tibetan population may impaired the power of results.
- Subgroup analysis maybe unavailable to perform due to the low quality of literature and missing confounding data, such as sex and age.

## 1 Introduction

*Helicobacter pylori* (Hp) have been categorized as a class-I carcinogenic pathogen of gastric cancer according to the World Health Organization.^[1]^ Solid evidence from epidemiologic studies have identified association between *Hp* infection and the progression of precancerous gastric lesions and development of gastric cancer.[^2^,^3^,^4^,^5^,^6^] Hp was approved to increase the risk of precancerous lesion atrophic gastritis in low incidence countries of gastric cancer.[^2^,^7^] As the third leading cause of deaths from cancer globally, Gastric cancer caused almost half of the estimated deaths in 2018 occurred in China.^[8]^ Many other gastrointestinal diseases caused by Hp infection have also brought huge economic and health care burden to China. So far, only Japan and South Korea established the nationwide organized screening of Hp infection and gastric cancer in the world,^[9]^ which improved the proportion of early diseases and resulted in better population survival of gastric cancer.^[10]^

In China, Hp infection prevalence ranges from 41.5% to 72.3%, varying with the ethnics and geographic area.[^11^,^12^] The overall prevalence of Hp infection was decreased to 56.2% in the 2002 to 2004 nationwide survey, but the highest prevalence of Hp infection was found in Tibet (84.6%).^[13]^ So it would be helpful to consider tailored screening and eradication strategy for Hp infection among ethnics.

To our knowledge, there was no systematic review or meta-analysis focused on the incidence of Hp between Tibetans and Hans. We will perform a systematic review and meta-analysis to compare the prevalence of Hp infection through meta-analyses using the available data on the incidence of Hp both in Tibetan and Han.

## 2 Methods

### 2.1 Reporting

This meta-analysis will be conducted according to the MOOSE 2000 statements,^[14]^ and a flow diagram will be drawn.^[15]^

### 2.2 Literature search

The PubMed will be comprehensively searched, using the following search terms, “((((Han[Title/Abstract]) OR Han nationality[Title/Abstract])) AND ((Tibet[Title/Abstract]) OR Tibetan)) AND (((HP[Title/Abstract]) OR H. pylori[Title/Abstract]) OR *Helicobacter Pylori*[Title/Abstract])”. No limits will be imposed on publication language, detection method, and observation period. A similar search strategy will be applied in searching published literatures in Web of Science, Technology Periodical Database (VIP), China National Knowledge infrastructure (CNKI), and WanFang databases.

### 2.3 Eligibility

Any type of cohort study, cross-sectional study, or case-control study conducted in China can be considered. Those studies that simultaneously reported the prevalence of Hp both in Tibetans and Hans is eligible. No restrictions on Hp detection method (breath test, biopsy, serological test, or stool test), publication language, and period of study conduction.

### 2.4 Selection and assessment

Two reviewers will separately screen the titles/abstracts, and identify the potentially eligible full-texts, according to inclusion and exclusion criteria. All of the included literatures will be confirmed by full-text review. Any disagreements on study selection will be resolved by consensus with a third reviewer. The Newcastle-Ottawa scale will be used to assess all the included studies.^[16]^ We will report the study selection process according to the Preferred Reporting Items for Systematic Reviews and Meta-Analyses (PRISMA) guidelines.

### 2.5 Data extraction

Two researchers will extract the data independently and check each other for accuracy. The following data of the selected studies will be extracted: publication year, sample size, sample region, Hp detection method, average age, sex proportion, and symptoms of upper gastrointestinal tract presenting or not. The subtotal of Tibetans or Hans, and the corresponding event numbers of Hp positivity will be extracted, too. Any discrepancies will be resolved by discussion between authors.

### 2.6 Statistics

The STATA 12.0 softwares, the Cochrane Reviewer Manager (RevMan) 5.3, and the PASS 11 will be used for statistical analysis.[^17^,^18^,^19^] The Mantel-Haenszel test or the DerSimonian-Laird test will be used for fixed or random model respectively, and 2-sided *P* values for the pooled ORs < .05 will be considered as statistical significance. The pooled prevalence of Hp infection in Tibetans and Hans will be combined in meta-analysis for rate, with 95% confidence intervals (CIs). We will calculate the pooled odds ratios (ORs) and their 95% CIs for Hp prevalence between Tibetans and Hans by fixed or random effect model. I-square will be estimated to test the statistical heterogeneity of the included studies. If the *P* values of heterogeneity test <.1, we will use a random-effects model for the calculation. Both the continuity corrected Begg rank correlation test and Egger linear regression test will be used.^[20]^ Any *P* value < 0.05 of Begg or Egger test will be considered as significance of publication bias. In Egger test, the intercept and its 95% CI will be estimated. Funnel plots will be drawn by the STATA 12.0 software to evaluate the publication bias.^[18]^ L’Abbé plot and Galbraith plot will be used to observe the heterogeneity. The power (1–β) will be estimated by the PASS 11 software for an individual study.^[19]^ The category of 2 independent proportions to test inequality will be selected, and parameter module of proportions will be used for calculation.^[21]^ Two-sided *Z* test (pooled) will be provided with α = 0.05. The leave-one-out method will be applied for those meta-analyses pooling at least 2 studies in sensitivity analysis. Additional sensitivity analysis will be performed according to exclude the studies with the power <0.70, or <0.80.

### 2.7 Ethics

The ethical approval will not be required due to the nature of literature-based research.

### 2.8 Registration

This study has been registered in the PROSPERO International Prospective Register of Systematic Reviews supported by the National Institute for Health Research of the National Health Service (NHS), UK (registration number: CRD42019121192).

## 3 Discussion

There has been a proven association between Hp infection and gastric cancer. Eradicating Hp could reduce the incidence of gastric cancer among healthy asymptomatic infected Asian individuals according to a systematic review and meta-analysis including randomized controlled trials from China, Japan, USA, and UK.^[22]^ The prevalence of Hp infection among Tibetans in China was also higher than the prevalence of the ethnic minority in Vietnam (38.1%) and Thailand (54.5%).[^23^,^24^] The average prevalence of Hp infection in China between 1983 and 2013 was 55%.^[25]^ It is of great importance to reduce the incidence of gastric cancer in certain specific population through screening and eradication of Hp.

As we know, the current study will be the first meta-analysis to compare the prevalence of Hp infection between Tibetan and Han ethnics. The results of our study may be informative for developing a tailored strong Hp infection screening and eradication strategy targeting Tibetans. Meanwhile, this study has been registered on PROSPERO, which makes it more transparent and trustworthy. Of course, there are limitations in our study, we hope to make a more comprehensive, detailed and further study in the future.

## Acknowledgments

The authors thank the substantial work of Volunteer Team of Gastric Cancer Surgery (VOLTGA), West China Hospital, Sichuan University, China.

## Author contributions

**Conceptualization:** Si-Yu Duan, Wei-Han Zhang.

**Data curation:** Dan Bai, Kai Liu, An-Mo Wang.

**Methodology:** Kai Liu, An-Mo Wang.

**Software:** Dan Bai.

**Supervision:** Jian-Kun Hu.

**Validation:** Dan Bai, Kai Liu, An-Mo Wang, Si-Yu Duan, Wei-Han Zhang, Jian-Kun Hu.

**Writing – original draft:** Dan Bai, Kai Liu, Si-Yu Duan.

**Writing – review & editing:** Wei-Han Zhang.

Dan Bai orcid: 0000-0002-0851-1847.
